# CXCL16 regulates cisplatin-induced acute kidney injury

**DOI:** 10.18632/oncotarget.9386

**Published:** 2016-05-15

**Authors:** Hua Liang, Zhengmao Zhang, Liqun He, Yanlin Wang

**Affiliations:** ^1^ Selzman Institute for Kidney Health and Section of Nephrology, Department of Medicine, Baylor College of Medicine, Houston, Texas, United States of America; ^2^ Department of Anesthesiology, Affiliated Foshan Hospital of Sun Yat-Sen University, Foshan, China; ^3^ Section of Nephrology, Department of Medicine, Shuguang Hospital, Shanghai, China; ^4^ Center for Translational Research on Inflammatory Diseases and Renal Section, Michael E. DeBakey Veterans Affairs Medical Center, Houston, Texas, United States of America

**Keywords:** acute kidney injury, cisplatin, chemokines, cytokines, apoptosis

## Abstract

The pathogenesis of cisplatin-induced acute kidney injury (AKI) is characterized by tubular cell apoptosis and inflammation. However, the molecular mechanisms are not fully understood. We found that CXCL16 was induced in renal tubular epithelial cells in response to cisplatin-induced AKI. Therefore, we investigated whether CXCL16 played a role in cisplatin–induced tubular cell apoptosis and inflammation. Wild-type and CXCL16 knockout mice were administrated with vehicle or cisplatin at 20 mg/kg by intraperitoneal injection. CXCL16 knockout mice had lower blood urea nitrogen and less tubular damage following cisplatin-induced AKI as compared with wild-type mice. Genetic disruption of CXCL16 reduced tubular epithelial cell apoptosis and decreased caspase-3 activation. Furthermore, CXCL16 deficiency inhibited infiltration of macrophages and T cells into the kidneys following cisplatin treatment, which was associated with reduced expression of the proinflammatory cytokines in the kidneys. Taken together, our results indicate that CXCL16 plays a crucial role in the pathogenesis of cisplatin–induced AKI through regulation of apoptosis and inflammation and maybe a novel therapeutic target for cisplatin-induced AKI.

## INTRODUCTION

Cisplatin (cis-Diamminedichloroplatinum II) is one of the most effective and widely used chemotherapeutic agents for the treatment of a variety of solid tumors [1]. One of the major side effects of cisplatin is acute kidney injury (AKI), which often limits the clinical utility of cisplatin [2, 3]. Furthermore, Cisplatin-induced AKI is associated with high morbidity and mortality [4]. The mechanism underlying cisplatin-induced AKI is complex and has not been fully elucidated, which involves in tubular necrosis/apoptosis, mitochondrial oxidative stress, and inflammation [5-8]. Therefore, an improved knowledge of the pathogenesis cisplatin-induced AKI is crucial for developing effective therapeutic strategy to prevent cisplatin-induced AKI and improve survival in cancer patients receiving cisplatin-based chemotherapy.

CXC chemokine ligand 16 (CXCL16) is a recently discovered cytokine belonging to the CXC chemokine family [9]. There are two forms of CXCL16. The soluble form generated by its cleavage at the cell surface functions as a chemoattractant to recruit circulating cells. The transmembrane form has a transmembrane structure that functions as an adhesion molecule for CXCR6-expressing cells and also serves as a scavenger receptor for oxidized lipoprotein and bacteria. CXCL16 is expressed at a low level in epithelial cells in the normal kidney and play a crucial role in regulating inflammation and tissue injury [10, 11]. We have recently demonstrated that CXCL16 contributes to chronic renal injury and fibrosis by recruiting fibrocytes, macrophages and T cells into the kidney [12, 13]. However, the role of CXCL16 in cisplatin-induced AKI remains unclear. In the present study, we found that CXCL16 is upregulated in the kidney in response to cisplatin-induced AKI. Therefore, we investigated the role of CXCL16 in experimental cisplatin-induced AKI by using CXCL16 knockout (KO) mice. Our results have shown that genetic disruption of CXCL16 protects the kidney from cisplatin-induced AKI through inhibiting apoptosis and inflammation.

## RESULTS

### CXCL16 is induced in cisplatin-induced AKI

**Figure 1:**
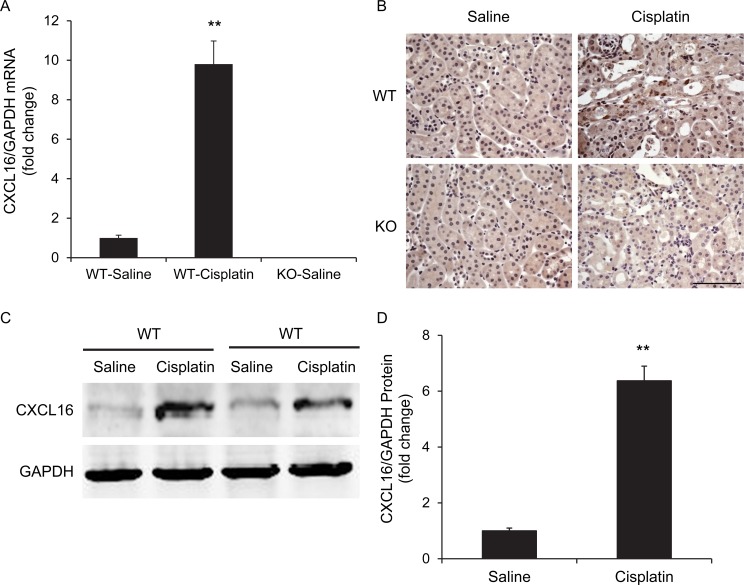
CXCL16 is upregulated in the kidney after cisplatin-induced acute kidney injury **A.** CXCL16 mRNA is induced markedly in kidneys of WT mice after cisplatin-induced AKI compared with vehicle control mice. **P < 0.01 *vs*. WT saline controls. *n* = 6 in each group. **B.** Representative photomicrographs of kidney sections stained for CXCL16 (brown) and counterstained with hematoxylin (blue). Scale bar: 50μm. **C.** Representative western blot shows CXCL16 protein levels in the kidneys of WT mice at 72 hours after vehicle or cisplatin treatment. **D.** Quantitative analysis of CXCL16 protein expression in the kidneys of WT mice. **P < 0.01 *vs*. WT saline controls. *n* = 6 in each group.

We first determined if CXCL16 is induced in the kidney in a mouse model of AKI induced by cisplatin. Using real-time reverse transcription-PCR (RT-PCR), we found that the mRNA levels of CXCL16 were upregulated significantly in the kidneys of cisplatin-treated wild-type (WT) mice compared with vehicle controls (Figure 1A). Of note, CXCL16 mRNA was not detected in CXCL16 KO mice, which confirms complete gene inactivation of CXCL16 in the KO mice. Immunohistochemical staining revealed that CXCL16 protein was expressed at a low level in epithelial cells in control kidneys and was markedly induced in the kidneys of cisplatin-treated WT mice (Figure 1B). No positive staining for CXCL16 was detected in KO mice, confirming the disruption of CXCL16 gene. In agreement with the immunohistochemical findings, Western blot analysis showed that the protein levels of CXCL16 were increased in injured kidneys of WT mice compared with control kidneys (Figure 1C and 1D).

### CXCL16 KO mice are protected from AKI

**Figure 2:**
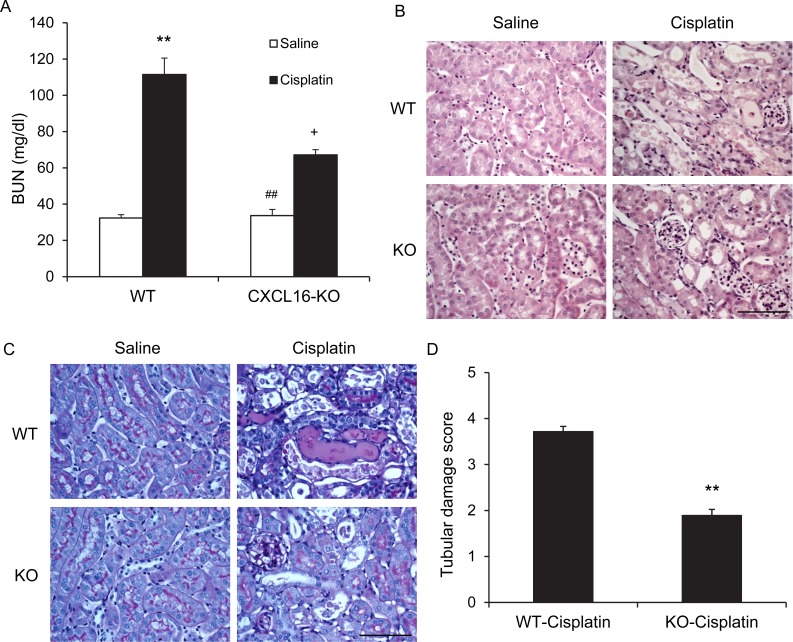
Genetic deficiency of CXCL16 protects kidney against cisplatin-induced injury **A.** Effect of CXCL16 deficiency on serum urea nitrogen in WT and CXCL16-KO mice at 72 hours after cisplatin or saline treatment. ^**^P < 0.01 *vs*. WT Saline, ^##^ *P* < 0.01 *vs*. CXCL16 KO cisplatin; ^+^ *P* < 0.05 *vs*. WT cisplatin. *n* = 6 in each group. **B.** Representative photomicrographs of hematoxylin and eosin staining for kidney sections of WT and CXCL16 KO mice at 72 hours after cisplatin or saline treatment. Scale bar: 50μm. **C.** Representative photomicrographs of Periodic Acid Schiff staining for kidney sections of WT and CXCL16 KO mice at 72 hours after cisplatin or saline treatment. Scale bar: 50μm. D. Quantitative assessment of tubular damage in WT and CXCL16 KO mice at 72 hours after cisplatin treatment. **P < 0.05 *vs*. WT cisplatin. *n* = 6 in each group.

To investigate the role of CXCL16 in the pathogenesis of cisplatin-induced AKI, WT and CXCL16 KO mice were treated i.p. with vehicle or cisplatin at 20 mg/kg. WT mice developed renal dysfunction as reflected by markedly elevation of blood urea nitrogen at 72 h after cisplatin treatment. Renal function was relatively preserved in CXCL16 KO mice with blood urea nitrogen markedly lower than WT mice (Figure 2A). Consistent with the preservation of kidney function in CXCL16 KO mice following cisplatin treatment, there was substantial reduction in histological injury of the kidneys as evidenced by less tubular epithelial cell injury, tubular dilation, and intratubular cast formation in CXCL16 KO mice (Figure 2B, 2C, and 2D).

### CXCL16 deficiency protects against apoptotic cell death

**Figure 3:**
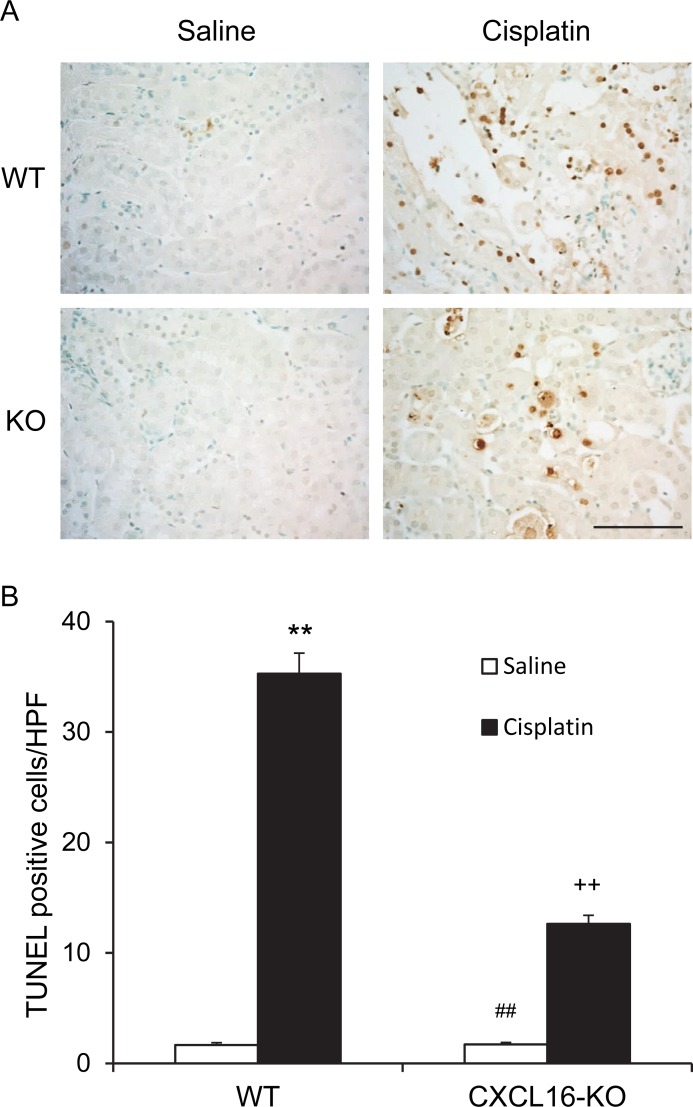
CXCL16 deficiency protects tubular epithelial cells from apoptosis in kidney of cisplatin-induced injury **A.** Representative photomicrographs of kidney sections stained for apoptotic cells (brown) and counterstained with methylgreen (green) in WT and CXCL16 KO mice at 72 hours after cisplatin or saline treatment. Scale bar: 50μm. **B.** Quantitative analysis of apoptotic cells in kidneys of WT and CXCL16 KO mice after cisplatin or saline treatment. **P < 0.01 *vs*. WT saline; ^##^*P* < 0.01 *vs*. CXCL16-KO cisplatin; ^++^*P* < 0.01 *vs*. WT cisplatin. *n* = 6 in each group. HPF, high power field; TUNEL, terminal transferase dUTP nick-end labeling.

**Figure 4:**
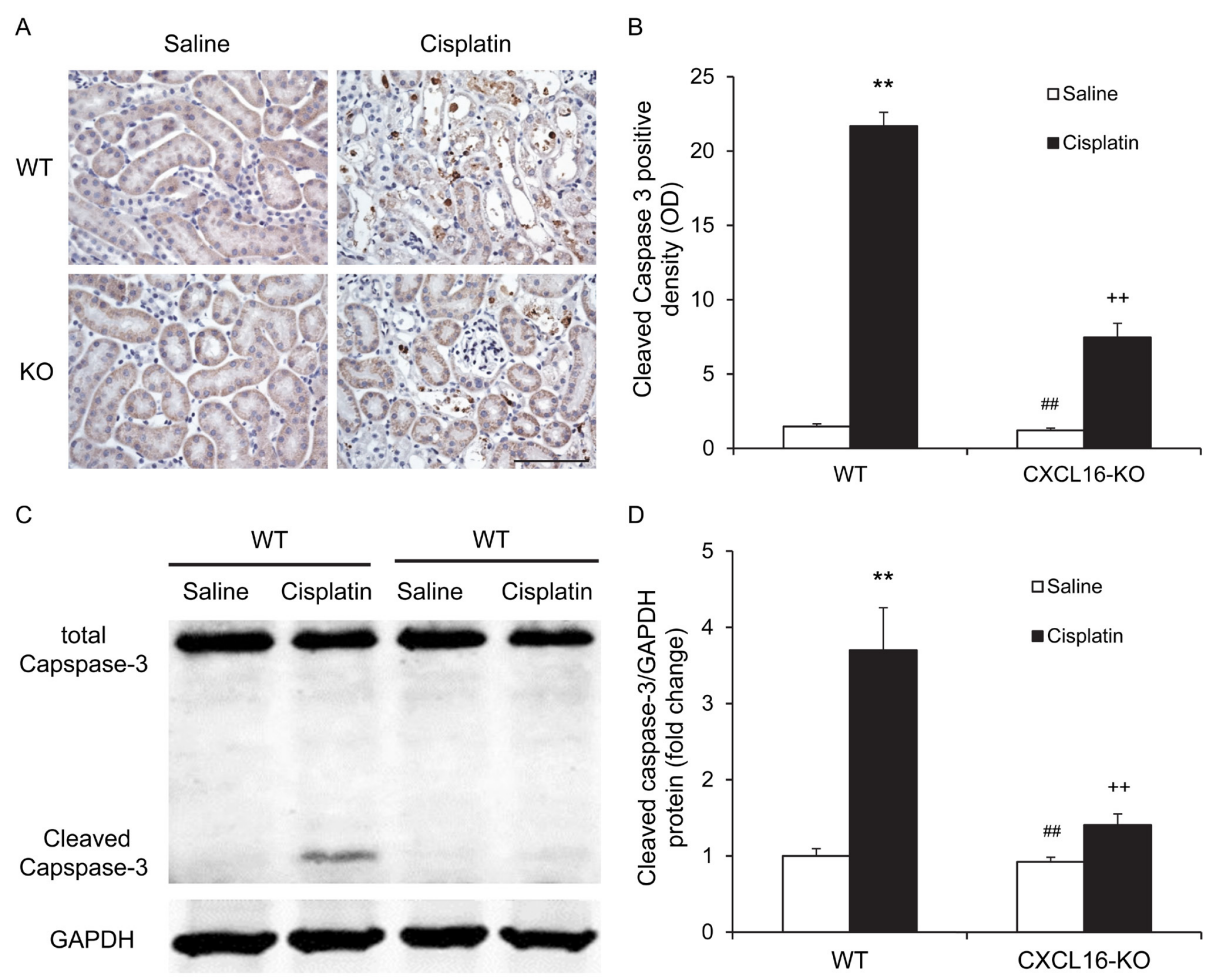
CXCL16 deficiency inhibits Caspase-3 activation in tubular epithelial cells **A.** Representative photomicrographs of kidney sections stained for cleaved caspase-3 (brown) and counterstained with hematoxylin (blue) in WT and CXCL16 KO mice at 72 hours after cisplatin or saline treatment. Scale bar: 50μm. **B.** Quantitative analysis of cleaved Caspase-3 expression in kidneys of WT and CXCL16 KO mice after cisplatin or saline treatment. **P < 0.01 *vs*. WT saline; ^##^*P* < 0.01 *vs*. CXCL16 KO cisplatin; ^++^*P* < 0.01 *vs*. WT cisplatin. *n* = 6 in each group. **C.** Representative western blots show cleaved Caspase-3 protein expression in kidneys of WT and CXCL16 KO mice after cisplatin or saline treatment. **D.** Quantitative analysis of cleaved Caspase-3 protein expression in kidneys of WT and CXCL16 KO mice after cisplatin or saline treatment. **P < 0.01 *vs*. WT saline; ^#^*P* < 0.05 *vs*. CXCL16 KO cisplatin; ^+^*P* < 0.05 *vs*. WT cisplatin. *n* = 6 in each group. HPF, high power field; GAPDH, glyceraldehyde-3-phosphate dehydrogenase.

Recent evidence indicate that tubular cell apoptosis contributes to the pathogenesis of cisplatin-induced AKI [14, 15]. Therefore, we examined the extent of cisplatin-induced tubular epithelial cell apoptosis in both WT and CXCL16 KO mice. Using terminal transferase dUTP nick-end labeling assay, we observed that the number of tubular apoptotic cells in kidneys of WT mice following cisplatin treatment was increased significantly, whereas the number of tubular apoptotic cells was markedly reduced in kidneys of CXCL16 KO mice treated with cisplatin (Figure 3A and 3B). Caspase-3 is a key effector caspase that initiates degradation of the cell in the final stages of apoptosis [16]. We next investigated the effect of CXCL16 deficiency on caspase-3 activation. Immunohistochemical staining showed that cleaved caspase-3 was induced in kidney tubular epithelial cells of WT mice following cisplatin treatment. In contract, disruption of CXCL16 significantly reduced the levels of cleaved caspase-3 in kidney tubular epithelial cells following cisplatin treatment (Figure 4A and 4B). In agreement with these findings, western blotting analysis using antibody against cleaved Caspase-3 revealed that the levels of active Caspase-3 were markedly increased in kidneys of WT mice as compared with vehicle control mice. Whereas, the levels of cleaved caspase-3 in kidneys of CXCL16 KO mice after cisplatin treatment was significantly lower than that in WT mice (Figure 4C and 4D). These data indicate that CXCL16 deficiency prevents Caspase-3 activation in the kidney following cisplatin treatment.

### CXCL16 deficiency impairs inflammatory cell infiltration

**Figure 5:**
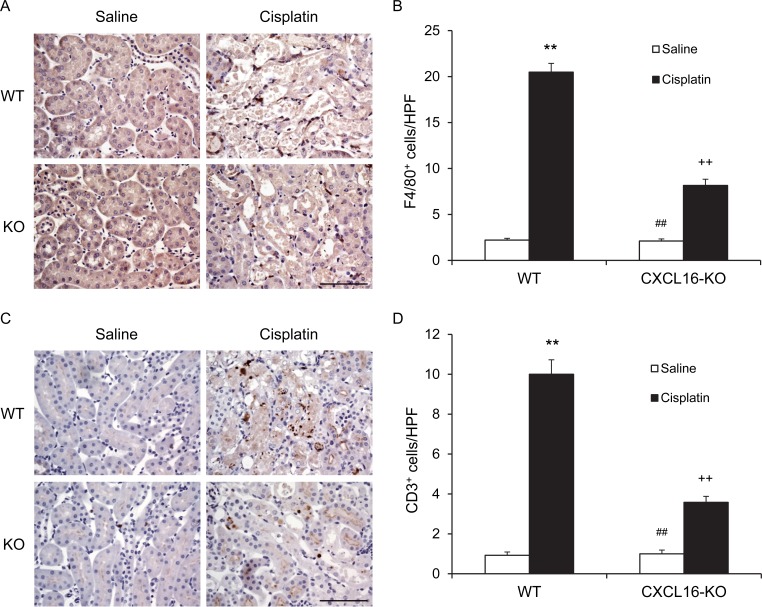
CXCL16 deficiency inhibits infiltration of macrophages and T cells in the kidney after cisplatin treatment **A.** Representative photomicrographs of kidney sections stained for F4/80 (brown) and counterstained with hematoxylin (blue) in kidneys of WT and CXCL16 KO mice after cisplatin or saline treatment. **V.** Quantitative analysis of F4/80^+^ macrophages in kidneys of WT and CXCL16 KO mice after cisplatin or saline treatment. **P < 0.01 *vs*. WT saline; ^##^*P* < 0.01 *vs*. CXCL16 KO cisplatin; ^++^*P* < 0.01 *vs*. WT cisplatin. *n* = 6 in each group. **C.** Representative photomicrographs of kidney sections stained for CD3 (brown) and counterstained with hematoxylin (blue) in kidneys of WT and CXCL16 KO mice after cisplatin or saline treatment. **D.** Quantitative analysis of CD3^+^ T cells in kidneys of WT and CXCL16 KO mice after cisplatin or saline treatment. **P < 0.01 *vs*. WT saline; ^##^*P* < 0.01 *vs*. CXCL16 KO cisplatin; ^++^*P* < 0.01 *vs*. WT cisplatin. *n* = 6 in each group. HPF, high power field.

Inflammatory cells have an important role in the pathogenesis of cisplatin-induced AKI [17]. To examine if CXCL16 is involved in the regulation of inflammatory cell infiltration into the kidney, kidney sections were stained for F4/80, a macrophage marker, and CD3, a lymphocyte marker. Significant infiltration of macrophages and T cells was observed in the kidneys of cisplatin-treated WT mice compared with vehicle-treated control group (Figure 5). In comparison, CXCL16 deficiency significantly suppressed the infiltration of macrophages and T cells into the kidneys after cisplatin treatment (Figure 5). These data indicate that CXCL16 promotes inflammatory cell infiltration into the kidney during cisplatin-induced AKI.

### CXCL16 deficiency suppresses inflammatory cytokine expression

**Figure 6:**
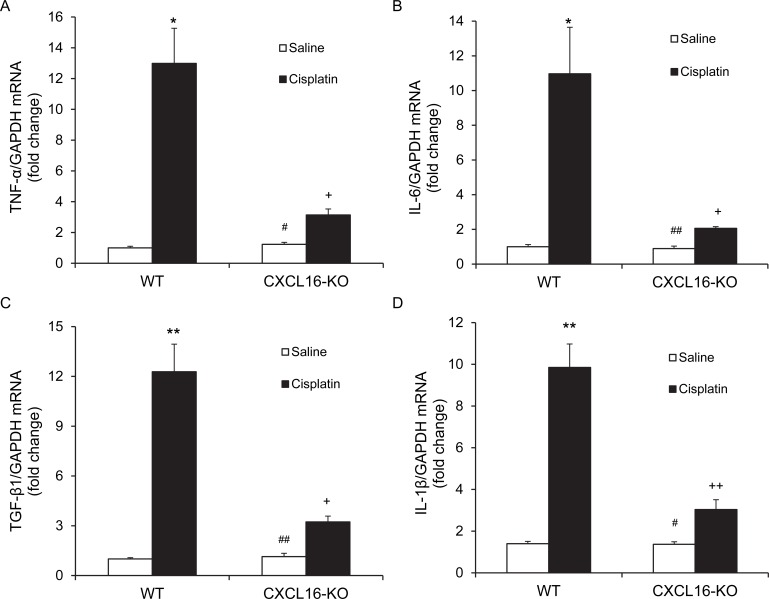
CXCL16 deficiency suppresses gene expression of proinflammatory molecules in the kidney after cisplatin treatment **A.** Quantitative analysis of TNF-α mRNA expression in kidneys of WT and CXCL16 KO mice after cisplatin or saline treatment. *P < 0.05 *vs*. WT CON; ^#^*P* < 0.05 *vs*. CXCL16 KO cisplatin; ^+^*P* < 0.05 *vs*. WT cisplatin. *n* = 6 in each group. **B.** Quantitative analysis of IL-6 mRNA expression in kidneys of WT and CXCL16 KO mice after cisplatin or saline treatment. *P < 0.05 *vs*. WT saline; ^##^*P* < 0.01 *vs*. CXCL16 KO cisplatin; ^+^*P* < 0.05 *vs*. WT cisplatin. *n* = 6 in each group. **C.** Quantitative analysis of TGF-β1 mRNA expression in kidneys of WT and CXCL16-KO mice after cisplatin or saline treatment. **P < 0.01 *vs*. WT saline; ^##^*P* < 0.01 *vs*. CXCL16 KO cisplatin; ^+^*P* < 0.05 *vs*. WT cisplatin. *n* = 6 in each group. **D.** Quantitative analysis of IL-1β mRNA expression in kidneys of WT and CXCL16 KO mice after cisplatin or saline treatment. **P < 0.01 *vs*. WT saline; ^#^*P* < 0.05 *vs*. CXCL16 KO cisplatin; ^++^*P* < 0.01 *vs*. WT cisplatin. *n* = 6 in each group. GAPDH, glyceraldehyde-3-phosphate dehydrogenase.

We next examined the effect of CXCL16 deficiency on the expression of known proinflammatory cytokines that are involved in the pathogenesis of cisplatin-induced AKI [18, 19]. The results of RT-PCR revealed that the mRNA levels of tumor necrosis factor a (TNF-a), interleukin 6 (IL-6), transforming growth factor β1 (TGF-β1), and interleukin 1β (IL-1β) were increased significantly in kidneys of WT mice after cisplatin administration as compared with vehicle controls (Figure 6). In comparison, the upregulation of TNF-a, IL-6, TGF-β1, and IL-1β was markedly attenuated in kidneys of CXCL16 KO mice (Figure 6). These results indicate that CXCL16 plays a role in promoting inflammatory cytokine expression in the kidney during cisplatin-induced AKI.

## DISCUSSION

Cisplatin is a widely prescribed chemotherapy agent for the treatment of a variety of cancer [1]. The development of nephrotoxicity often limits its clinical use [2, 3]. At present, there is no effective therapy for cisplatin-induced AKI. Therefore, a better understanding of the pathogenesis of cisplatin-induced AKI is much needed to protect the normal cells while unshielding cancer cells [20]. CXCL16 is a chemokine that plays an important role in regulating inflammation, tissue injury, and fibrosis [10]. However, its role in cisplatin-induced AKI remains unknown. In the current study, we have demonstrated the following: (1) CXCL16 is induced in the kidney in response to cisplatin-induced AKI; (2) Genetic disruption of CXCL16 protects against cisplatin-induced AKI and tubular epithelial cell apoptosis; (3) Genetic disruption of CXCL16 reduces inflammatory cell infiltration and proinflammatory cytokine production in the kidney. These results indicate that CXCL16 has a key role in the pathogenesis of cisplatin-induced AKI by regulating apoptosis and inflammation.

CXCL16 was originally described as a scavenger receptor for phosphatidylserine and oxidized low-density lipoprotein termed SR-PSOX [21]. Recent studies have shown that CXCL16 regulates cell adhesion and migration through interaction with its receptor - CXCR6 [10, 22, 23]. CXCL16 has a proinflammatory effect on renal proximal tubular cells and potentiates TWEAK-induced inflammatory responses [24]. Our previous studies have shown that CXCL16 is upregulated in the kidney following unilateral ureteral obstruction and angiotensin-induced renal injury, and genetic disruption of CXCL16 attenuates renal fibrosis and preserves kidney function [12, 13]. In the present study, we have demonstrated that CXCL16 level is increased in the kidney following cisplatin administration. Subsequently, we explore the role of CXCL16 in cisplatin-induced AKI using CXCL16 KO mice. Our results have shown that CXCL16 deficiency preserves renal function and ameliorates tubular damage after cisplatin treatment. These data indicate that CXCL16 promotes cisplatin-induced AKI.

Apoptosis of tubular epithelial cells contribute to cisplatin-induced AKI, and inhibition of apoptosis may be therapeutic strategy for cisplatin-induced AKI [25, 26]. In the current study, cisplatin significantly increased the number of TUNEL-positive cells in WT mice, which was markedly reduced by disruption of CXCL16. The Caspase family of cysteine proteases involved in the initiation and execution of the mammalian apoptotic cell death program. Caspase-3 is formed from a 32 kDa zymogen that is cleaved into active 17 kDa subunits by death ligand and mitochondrial pathways. Caspase-3 activation has been considered as a key mechanism underlying the pathogenesis of cisplatin-induced apoptotic cell death in tubular epithelial cells [27, 28]. In the present study, we have examined the effect of CXCL16 disruption on cisplatin-induced caspase-3 activation in the kidneys. Our results have demonstrated that caspase-3 is significantly activated in the kidneys after cisplatin treatment in WT mice, whereas caspase-3 activation is markedly reduced in CXCL16 KO mice in response to cisplatin treatment. These data indicate that CXCL16 deficiency inhibits caspase-3 activation and apoptosis.

Inflammation plays an important role in the pathogenesis of cisplatin nephrotoxicity [7, 29, 30]. Inflammatory cells such as macrophages and T cells are known to infiltrate the kidney tissue and play a key role in the development of cisplatin-induced AKI [30]. However, the underlying mechanisms are not completely understood. In our previous study, we have shown that CXCL16 is expressed by injured tubular epithelium and functions as a potent chemoattractant for inflammatory cells in a hypertensive renal injury model, and genetic disruption of CXCL16 attenuates the inflammatory response by inhibiting accumulation of macrophages and T cells in the kidneys [12]. In the present study, we have demonstrated that CXCL16 KO mice exhibit a reduced infiltration of macrophages and T cells into the kidney after cisplatin treatment compared with WT mice. There results indicate that CXCL16 promotes cisplatin-induced AKI through recruiting inflammatory cells into the kidney. The molecular signaling mechanisms underlying CXCL16-induced inflammatory cell migration are not known. One potential candidate is AKT/mammalian target of rapamycin (mTOR) signaling pathway because CXCL16 has been reported to promotes prostate cancer progression by activating the AKT/mTOR signaling pathway [31], which is abolished by rapamycin, a mTOR inhibitor and an anti-aging and anti-cancer drug [32, 33]. Whether rapamycin has a protective role in cisplatin-induced kidney injury requires further investigation.

The proinflammatory nature of cisplatin-induced AKI has been well established [6, 18]. Recent studies have shown that proinflammatory cytokines such as TNF-α, IL-6, TGF-β1, and IL-1β contribute to the development of cisplatin-induced AKI [29]. In the present study, we have examined the mRNA expression of TNF-α, IL-6, TGF-β1, and IL-1β in the kidney following cisplatin administration. Our results reveal that genetic disruption of CXCL16 suppresses the mRNA expression of the proinflammatory molecules - TNF-α, IL-6, TGF-β1, and IL-1β in the kidney following cisplatin treatment. Using an RNA interference screen, Zynda et al. have identified novel genes including TNFAIP6 whose suppression improves survival of kidney epithelial cells [34]. It will be interesting to determine if these genes are regulated by CXCL16 in the kidney after cisplatin treatment.

In summary, our study has shown that CXCL16 plays a crucial role in the pathogenesis of cisplatin-induced AKI. In response to cisplatin exposure, chemokine CXCL16 recruits circulating inflammatory cells into the kidney leading to production of inflammatory molecules and subsequent tubular epithelial cell apoptosis and kidney dysfunction. These data indicate that inhibition of CXCL16 could represent a novel therapeutic approach for the treatment of cisplatin-induced AKI.

## MATERIALS AND METHODS

### Animals

WT C57BL/6 mice were purchased from the Jackson Laboratory and CXCL16 KO mice on a C57BL/6 background were a generous gift from Dr. Shuhua Han at Baylor College of Medicine [12, 13]. Mice were bred and maintained in the animal care facility of Baylor College of Medicine and had access to food and water ad libitum. All animal procedures were in accordance with national and international animal care and ethical guidelines and have been approved by the institutional animal welfare committee. Cisplatin was dissolved directly in 0.9% saline for 1mg/ml. Male WT mice and CXCL16 KO mice, 8-10 weeks old, weighting 20-30 g, were administrated cisplatin (20 mg/kg) or saline by intraperitoneal (i.p) injection. Animals were sacrificed at 72 h after cisplatin injection. Kidneys were perfused with PBS and harvested.

### Renal function

Blood urea nitrogen was detected fluorometrically as described [12, 22, 35, 36].

### Renal morphology

Kidney tissue was fixed in 10% buffered formalin, embedded in paraffin, and cut at 4-μm-thick sections. After deparaffinization and rehydration, sections were stained with hematoxylin and eosin or periodic Acid Schiff (PAS). Tissue damage was examined in a blinded manner and scored according to the percentage of damaged tubules: 0, no damage; 1, <25% damage; 2, 25-50% damage; 3, 50-75% damage; and 4,>75% damage as reported [36, 37].

### Immunohistochemistry

Immunohistochemical staining was performed on paraffin sections. Antigen retrieval was performed with antigen unmasking solution (Vector Laboratories) or proteinase K. Endogenous peroxidase activity was quenched with 3% H_2_O_2_ for 10 min. After blocking with 5% normal serum, slides were incubated with primary antibodies in a humidified chamber overnight. After washing, slides were incubated with appropriate secondary antibodies and ABC solution sequentially according to the ABC kit (Vector Laboratories). Slides were then visualized by incubation in diaminobenzidine solution for an appropriate period of time. Nuclear staining was performed with hematoxylin. The slides were dehydrated, cleared, and mounted. The images from these slides were obtained and analyzed by NIS Element software (Nikon Instruments) with Nikon microscope image system (Nikon Instruments).

### Apoptosis detection

A terminal transferase dUTP nick-end labeling assay was performed to evaluate apoptosis using an ApopTag plus Peroxidase in situ Apoptosis Detection Kit (Millipore, Billerica, MA) according to the manufacturer's instruction. The number of terminal transferase dUTP nick-end labeling-positive cells per high-power field were counted and analyzed in a blinded manner [36].

### Real-time RT-PCR

Total RNA was extracted from the kidney tissues with TRIzol reagent (Invitrogen, Carlsbad, CA). Aliquots (1 μg) of total RNA were reverse transcribed using SuperScript II reverse transcriptase. Quantitative Real-Time PCR was performed using IQ SYBR green supermix reagent (Bio-Rad, Herculus, CA) with a Bio-Rad real-time PCR machine according to the manufacturer's instructions. The comparative Ct method (ΔΔCt) was used to quantify gene expression, and the relative quantification was calculated as 2^−ΔΔCt^. [12, 22, 36] The expression levels of the target genes were normalized to glyceraldehyde-3-phosphate dehydrogenase (GAPDH) level in each sample. The primer sequences were as follows: CXCL16 - forward 5′-ACCCTTGTCTCTTGCGTTCTTCCT-3′, reverse 5′-ATGTGATCCAAAGTACCCTGCGGT-3′; IL-6 - forward, 5′-AGGATACCACTCCCAACAGACCTG-3′, reverse, 5′-CTGCAAGTGCATCATCGTTGTTCA-3′; TNFα - forward, 5′-CATGAGCACAGAAAGCATGATCCG-3′, reverse, 5′-AAGCAGGAATGAGAAGAGGCTGAG-3′; IL-1β - forward, 5′-CTTCAGGCAGGCAGTATCACTCAT-3′, reverse, 5′-TCTAATGGGAACGTCACACACCAG-3′; TGF-β1 - forward, 5′-CAACAATTCCTGGCGTTACCTTGG-3′, reverse, 5′-GAAAGCCCTGTATTCCGTCTCCTT-3′; and GAPDH - forward, 5′-CCAATGTGTCCGTCGCGTGGATCT-3′, reverse, 5′-GTTGAAGTCGCAGGAGACAACC-3′.

### Western blot analysis

Protein was extracted using RIPA buffer containing cocktail proteinase inhibitors and quantified with a Bio-Rad protein assay. An equal amount of protein was separated on SDS-polycrylamide gels in Tris/SDS buffer system, and then transferred onto nitrocellulose membranes. The membranes were incubated with primary antibodies (CXCL16 and caspase 3) overnight, followed by incubation with appropriate fluorescence-conjugated secondary antibodies. The proteins of interest were analyzed using an Odyssey (LI-COR Bioscience, Lincoln, NE) IR scanner, and signal intensities were quantified using NIH Image/J software (National Institutes of Health, Bethesda, MD).

### Statistical analysis

Data were expressed as mean ± SEM. Two group comparisons were performed by Student's t test. Multiple group comparisons were performed by ANOVA followed by the Bonferroni procedure for comparison of means. A *P* value < 0.05 was considered statistically significant.
